# The Impact of Hotel Customer Engagement and Service Evaluation on Customer Behavior Intention: The Mediating Effect of Brand Trust

**DOI:** 10.3389/fpsyg.2022.852336

**Published:** 2022-04-26

**Authors:** Xi Chen, Yifan Wang, Xujie Lyu, Jinlong Zhang

**Affiliations:** ^1^Business School, Shandong Jianzhu University, Jinan, China; ^2^Business School, Heze University, Heze, China; ^3^Global Food Service Management, Woosuk University, Jeonju, South Korea

**Keywords:** service quality, brand trust, customer’s intention, hotel, customer engagement (CE)

## Abstract

Because of the COVID-19, the tourism industry has been greatly affected, especially the occupancy rate of hotel companies. This study analyzes the effects of customer engagement and service evaluation on brand trust and customer behavioral intention based on 437 valid questionnaires from Chinese economy hotel companies using SPSS and AMOS. The components of customer engagement are subdivided into five dimensions: identification, enthusiasm, attention, absorption and interaction, and the impact of these five dimensions on brand trust in the COVID- 19 is investigated. Finally, it verifies the influence of trust on customers’ word-of-mouth (WOM) intention and customers’ reuse intention. The results of this study not only enrich the research on customer engagement and service evaluation in marketing circles but also give some advice to hotel companies in the COVID-19 customer engagement and service evaluation that can enhance the trust of enterprises and promote the behavior intention of customers, which has certain practical reference value.

## Introduction

The outbreak and spread of the COVID-19 in 2020 have caused a great impact on the tourism industry in China, with the traditional tourism industry such as scenic spots, hotels and travel agencies facing great pressure on their operations. In the case of the hotel industry, the impact of the epidemic on hotels has continued to be felt since the second quarter of 2020, highlighted by a simultaneous decline in average daily room rates and occupancy rates, challenging the profitability of hotels, with some experts predicting that the epidemic may result in hotels closing down to weather the crisis (Chen, 2020). Due to the decrease in tourism travel and business mobility, economy hotels, which mainly serve the mass tourism travelers and small and medium-sized business travelers, are facing development difficulties. How economy hotels respond has become an important issue of concern for the industry and academia.

With the rapid development of the Internet, customers can easily interact with other customers or businesses through social media. As a result, the non-transactional behaviors of customers are also drawing more and more attention from enterprises. Companies are discovering that certain non-transactional behaviors of customers, such as positive word-of-mouth (WOM) and online reviews (Van Doorn et al., 2010), can have a positive impact on the company. At the same time, companies are aware that non-transactional customer behavior can have serious consequences if not managed properly, and thus the concept of customer engagement has emerged.

In the past few years, the research on customer engagement has been diverse and complex. The individual perceived costs and benefits have an impact on customer engagement (Van Doorn et al., 2010). Customer involvement and participation, such as customer involvement in the development of new products in a company, can have a positive effect on customer engagement (Vivek et al., 2014). Zhang et al. (2021) showed that relationship quality had a positive effect on customer engagement. The drivers of customer engagement behavior are based on organizational support theory, so both perceived customer support and customer commitment are antecedent variables of customer engagement (Wang, 2020). Customer engagement is positively influenced by interactivity and information quality (Islam et al., 2017). Customer engagement is a special value-driven relationship between customers and enterprises, which has a significant impact on the relationship between other customers and enterprises (Chan et al., 2014). The value-driven relationship between enterprises and customers can create value for enterprises, and may also weaken value (Dessart et al., 2015). It is a positive relationship between customer engagement and cognitive value (Algharabat et al., 2020). Zameer et al. (2018) conducted an empirical study using m-commerce as an example and showed that mobile electronic service quality had a direct positive impact on customer engagement, while age had a moderating effect in the middle. Although scholars have examined specific aspects of customer engagement in different contexts in previous studies, this study integrates psychological and behavioral perspectives to show that customer engagement is a result of both psychological and behavioral aspects. Currently, few studies have measured the components of customer engagement in a multidimensional manner.

The tourism industry has been hit hard by COVID-19 and hotel companies are experiencing an unprecedented decline in occupancy rates. What can be done to improve consumer engagement with hotel companies? How can we improve the service rating of hotel companies? How can hotel companies gain consumers’ trust in their brand? How do they generate WOM intention and reuse intention?

In this study, customers who have used economy hotels are used as the target group. The components of customer engagement are subdivided into five dimensions: identification, enthusiasm, attention, absorption and interaction, and the impact of these five dimensions on brand trust in the COVID-19 is investigated. In addition, this study divides service evaluation into three dimensions, namely service quality, perceived value, and customer satisfaction, and explores how to enhance consumers’ brand trust in hotels by improving their service quality, perceived value, and customer satisfaction, which in turn leads to WOM intentions and reuse intentions.

This study provides a theoretically sound scale for marketing, which scholars can use to further expand their understanding of customer engagement. In addition, the fundamental contribution of this study is to provide a theoretical foundation and empirical evidence to support the relationship between the emerging concept of customer engagement and key factors in the development of customer behavioral intentions. In addition to theoretical contributions, this study provides some practical insights into the practice of brand management. The development and validation of the Customer engagement Scale provide a valuable tool for economy hotels to effectively measure engagement with customer groups and rationalize marketing strategies during the COVID-19. This study is a useful reference for economy hotels to encourage customer engagement with the hotel or other customers and enhance trust in the company.

## Literature Review and Hypotheses Development

### The Stimulus-Organism-Response Framework

Originally, in Stimulus-Organism-Response (S-O-R) model, invented by Mehrabian and Russell (1974), an environment stimulus (S) results in an emotional response (O) thereby fostering a behavioral response (R). The S-O-R model is applied for the framework building of this research, and there are two reasons for choosing this model.

Firstly, its importance in retail settings has been articulated by various scholars from different areas such as decision to buy (Demangeot and Broderick, 2016; Lucia-Palacios et al., 2016), impulse buying (Chang et al., 2013), service fairness (Namkung and Jang, 2010), etc. Many S-O-R based research works in the marketing context confirm the relationship between emotional response and consumer response in terms of intention, purchase, consultation and return (Choi et al., 2011; Li et al., 2012). Especially the Chinese people who are collectivism dominated in the multicultural social environment would be more sensitive to various environmental cues (Shobeiri et al., 2018; Jiao et al., 2021). Secondly, within the S-O-R model, when consumers accept multisensory input from the external environment, such as COVID-19, the internal state of consumers will also affect their approach or avoidance actions (Eroglu et al., 2003). Some researchers use S-O-R model to explain consumption behaviors stimulated by various external environments, and some predictions have been made by using S-O-R model (Russell and Mehrabian, 1974; Shen et al., 2021).

In this study, the stimulus reflects the factors that determine the performance of hotels (service evaluation). This organism reflects consumers’ emotions and cognitive states (brand trust) and acts as an intermediary platform to produce specific behavioral outcomes (behavior intention). Therefore, the S-O-R model could be used to explain the relationship among customer engagement, service evaluation, brand trust, and behavior intention.

### Customer Engagement

For the concept of customer engagement, Kaplan et al. (2006) defined “engagement” as the psychological concept of employee participation and engagement in the work environment. After that, based on this research, many scholars applied the concept of “engagement” to sociology, psychology, pedagogy, and other fields (Brodie and Hollebeek, 2011; Guo et al., 2016). In the previous studies, customer engagement is defined as the emotional bond between the enterprise and customers (Rieger and Kamins, 2006), the interaction with customer participation (Nambisan, 2002; Wagner and Majchrzak, 2006), the spontaneous participation behavior through knowledge exchange among customers (Joshi and Sharma, 2004; Erat and Kavadias, 2006).

In the past few years, the perception of “customer engagement” contains psychological, behavioral and integrated dimensions. The psychological dimension considers customer engagement as the mental state or mental process of both cognitive and emotional aspects of the customer toward the company. Bowden (2009) introduced the first cognitive and affective-based measurement dimensions from a biased psychological dimension. Hollebeek (2011) described three dimensions of customer engagement: cognitive, emotional and behavioral. A type of WOM transfer, recommendation, C2C interaction, blogging, writing reviews and other similar activities by customers to a brand or company are typical of the behavioral dimension. Van Doorn et al. (2010) clearly pointed out that customer engagement was a manifestation of behavior that contained two main aspects of recommendation and review; Vivek (2009) proposed that customer engagement could be measured in three dimensions: conscious participation, social interaction, and enthusiasm in the behavioral dimension. The integrated dimension, on the other hand, integrates psychological and behavioral perspectives, pointing out that customer engagement is the result of both psychological and behavioral aspects. Dwivedi (2015) expressed it in a more directional way, dividing it into three dimensions: vigor, dedication, absorption (Dwivedi, 2015). Dessart et al. (2015) built on three dimensions that were further subdivided by subdividing the cognitive dimension into identification, absorption, and attention, the affective dimension into enthusiasm and enjoyment, and the behavioral dimension into interaction and learning. So et al. (2014) conceptualized customer engagement in the context of tourism branding into five dimensions enthusiasm, attention, absorption, interaction, and identification. In this study, from the psychological and behavioral multidimensional, and eventually referring to So et al. (2013)’s measurement dimensions, the customer engagement components are subdivided into five dimensions of identification, enthusiasm, attention, absorption, and interaction to study the impact of each customer engagement dimension on brand trust and consumer behavioral intention in the COVID-19. This method of measurement uses rooted theory and empirical testing to arrive at a relatively complete customer engagement measurement system that is consistent with management practice in the COVID-19 in China. The measurement system avoids the problem of crossover between variables in other scales, has a high degree of compatibility with the practice of economy hotels, and has a certain degree of operability.

Brodie and Hollebeek (2011) in the exploratory research of customer engagement behavior in virtual online brands confirmed that customer trust was the result variable of customer engagement. Brodie et al. (2011), while exploring the internal structure and formation system of customer engagement in online social media, think that customer engagement formed through five sub-processes: learning, sharing, advocating, socializing and co-development can have an impact on customer trust and customer commitment. Qiang et al. (2018) hold that customer trust refers to the subjective expectation of members of social groups for the potential value that knowledge sharing may bring, while customer engagement emphasizes the participation or input level of effective interaction among members in the process of knowledge dissemination and sharing. Therefore, in the process of knowledge interaction, members’ input in cognition, emotion, and behavior can increase their trust in the community. Based on the above discussion, the following research hypotheses are put forward:

**H1:** Customer engagement has a direct positive impact on brand trust.

Identification is essentially a perceptual and cognitive structure (Mael and Ashforth, 1992), which means identification matching. The concept of identification comes from the theory of social identification, which holds that self-concept consists of personal identification and consists of characteristics such as ability and interest (Ashforth and Mael, 1989). People develop social identification by dividing themselves and others into different social groups. When a person thinks that he/she is intertwined with the characteristics of this group, a sense of identification will arise. From the consumer’s point of view, identification is an individual’s perception of organizational unity or belonging (Bhattacharya and Sen, 2003). At the brand level, when consumers think that their self-image is consistent with the brand image, they will have an identification (Bagozzi and Dholakia, 2006). Therefore, identification, as an important dimension of consumer participation, is of great significance to customer engagement. Hence, we hypothesize:

**H1a:** Identification has a direct positive impact on brand trust.

Enthusiasm represents an individual’s level of intense excitement and interest in engagement focus (Vivek, 2009). Enthusiasm is considered to be a positive emotional state of work engagement and customer engagement. Enthusiasm is characterized by a strong sense of excitement (Bloch, 1986), which is a lasting and active state. Vivek (2009) also emphasizes the importance of enthusiasm and excitement and regards enthusiasm as a unique dimension to capture consumers’ strong excitement and focus. Hence, we hypothesize:

**H1b:** Enthusiasm has a direct positive effect on brand trust.

Previous studies have shown that attention is a key aspect of engagement. Attention is the duration of focus on work and mental focus (Rothbard, 2001). Highly engaged people tend to focus a lot of attention consciously or unconsciously on the people they are engaged in. Similarly, personal engagement is related to attention, connection, integration, and absorption of in-role performance (Kahn, 1992). Therefore, consumer attention is considered an important aspect of customer engagement. Hence, we hypothesize:

**H1c:** Attention has a direct positive impact on brand trust.

Absorption has been identified by many scholars as an important indicator of customer engagement (Hollebeek and Brodie, 2009). Absorption is to be so absorbed in something that time passes so quickly that a person is very detached from the role. Absorption is a high degree of attention and absorption that transcends sensory efficiency is a state of optimum experience (Schaufeli et al., 2002). In marketing, absorption represents effortless attention, loss of self-awareness, distortion of time, and inner enjoyment. Therefore, absorption is an important indicator of customer engagement. Hence, we hypothesize:

**H1d:** Absorption has a direct positive impact on brand trust.

Interaction refers to customers’ online and offline participation in brand activities or contact with other customers besides purchase. Interaction includes sharing and communicating thoughts and feelings about brand experience, which is an important part of customer engagement (Vivek, 2009). The main form of expression is through oral communication, recommendation, customer interaction, blogging, writing comments, and other ways to participate in company activities (Van Doorn et al., 2010). With the increase of participation intensity, the possibility of customers participating in these activities will also increase. Therefore, interactive customer engagement is an important dimension. Hence, we hypothesize:

**H1e:** Interaction has a direct positive impact on brand trust.

### Service Evaluation

In brand loyalty literature, service quality, satisfaction, and value are described as evaluative judgment variables (Butcher et al., 2001) or service evaluation variables (Lai et al., 2009), which directly depend on customers’ evaluation of actual service provision or service consumption experience. Although these variables are different concepts and represent the cornerstone of service brand loyalty, previous empirical studies have always found that these evaluation factors are interrelated (Cronin et al., 2000; Lai et al., 2009). Therefore, these well-established brand loyalty precedents can be collectively referred to as service evaluation variables, which are mainly determined by consumers’ perception of service experience, which is helpful to the formation of service brand loyalty.

In a large number of marketing literature, closely related structures are combined to form a higher level of abstraction. For example, a large number of research literature show that the overall evaluation of customers, such as overall satisfaction, perceived service quality, and perceived value usually has a strong statistical relationship, which is described as the halo effect (Crosby and Stephens, 1987) or multicollinearity (Rust et al., 1995).

This effect is considered to be the result of cognitive and memory processes, in which the overall assessment synthesizes many experiences and perceptions (Garbarino and Johnson, 1999). It is irrelevant whether researchers use customer satisfaction or service quality to determine quality return because these evaluation factors are similar in forming consumers’ views on service companies (Rust et al., 1995). In addition, Cowles and Crosby adopted a similar approach when proposing and testing the relationship quality model, in which different types of cumulative evaluations (such as trust and satisfaction) are combined to form a structure called relationship quality (Cowles and Crosby, 1990). Therefore, on this basis, it is conceptually appropriate to combine perceived service quality, perceived value, and customer satisfaction to form a higher-level service evaluation structure.

#### Service Quality

A widely studied antecedent of trust is service quality. Service quality is the judgment of consumers on the overall excellence or superiority of products (Zeithaml, 1988). Up to now, most descriptions of service quality in service environment are rooted in unconfirmed paradigm (Berry et al., 1985; Parasuraman et al., 1985; Zeithaml, 1988), which shows that the quality of service is determined by comparing expectations with performance. A review of the literature shows that service quality has several concepts (Lehtinen and Lehtinen, 1982; Ravald and Grönroos, 1996). However, the most widely used concept of service quality identifies reliability, responsiveness, assurance, empathy, and materiality as the five basic dimensions that consumers use to evaluate service quality (Zeithaml, 1988; Parasuraman et al., 1991).

When investigating the relationship between service quality and loyalty, researchers found that service quality directly determined customer loyalty to products or brands (Bitner, 1990; Zeithaml et al., 1996; Bloemer et al., 1999; Lee and Cunningham, 2001; Aydin et al., 2005; Rauyruen and Miller, 2007; Han et al., 2011; Cheng et al., 2012).

This relationship can be explained by the behavioral consequences model of service quality (Zeithaml et al., 1996), which assumes that high evaluation of service quality will lead to good behavior intention of customers, such as loyalty to service quality. This effect occurs because enhanced service quality helps consumers to develop good attitudes toward service providers and thus develop preference loyalty (Bloemer et al., 1999). Empirical evidence supports the impact of service quality on willingness to buy back (Rauyruen and Miller, 2007), willingness to recommend (Bloemer et al., 1998), and service loyalty (Caruana, 2002). Therefore, excellent service quality produces brand loyalty.

Besides having direct predictive power in explaining brand loyalty, service quality also indirectly affects brand loyalty through customer satisfaction (Butcher et al., 2001; Caruana, 2002; Olsen, 2002; Ball et al., 2004; Yu et al., 2005; Chiou and Droge, 2006; Han et al., 2011; Kim, 2011). The framework of Lazarus (1991) and Bagozzi (1992) provides a theoretical basis for indirect relations, which includes evaluation, emotional response, and coping.

This framework shows that consumers form an attitude toward the quality of products, brands, or stores by understanding the different characteristics of products, brands, or stores, thus generating a global emotional evaluation (i.e., satisfaction). This emotional evaluation then becomes the tendency to guide the final brand selection and loyalty (Olsen, 2002), thus forming a sequential chain effect of service quality, customer satisfaction, and brand loyalty in the development of loyalty. Thus, service quality has both direct and indirect effects on loyalty (through satisfaction) (Cronin et al., 2000; Sivadas and Baker-Prewitt, 2000; Lee et al., 2004; Petrick, 2004a; Eiselt and Marianov, 2009).

Hardin (2000) defines “trust” as giving one’s interests to others without damaging one’s interests, and emphasizes that trust is a variable of uniting organizations. The research results on hotel websites show that the influence of service quality on trust is obvious (Forgas et al., 2012). Trust of enterprises in websites directly affects the service quality of personal information protection, transaction stability, information, and interactivity (Walczuch and Lundgren, 2004). Thus, hypothesis 2a is as follows:

**H2a:** Service quality has a direct positive impact on brand trust.

#### Perceived Value

Perceived value is also considered a key driver of loyalty. The most conceptual definition of perceived value is based on Zeithaml’s statement that value represents “the consumer’s overall assessment of product utility based on perceptions received and given” (Zeithaml, 1988, p. 14). From this perspective, the view of value reflects a reasonable trade-off between the cost and benefit of using a product or service (Zeithaml, 1988; Dodds et al., 1991; Grewal et al., 1998; Cronin et al., 2000; Anderson and Srinivasan, 2003; Petrick, 2004a). When proposing the relationship between perceived value and brand loyalty, Sirdeshmukh et al. (2002) describe value as a superior consumer goal that regulates consumer behavior at the level of behavior intention of loyalty (Sirdeshmukh et al., 2002).

Consumers are expected to regulate their behavior to achieve this goal, so as long as the purchase provides higher value, they show loyal behavior intention. In addition, previous studies have shown that perceived value affects revisit intention (Oh, 1999; Petrick, 2004b; Kim et al., 2008), Commitment (Pura, 2005; Han et al., 2011) and Brand Loyalty (Sirdeshmukh et al., 2002; Chen and Chen, 2010). Therefore, the review of loyalty literature indicates that perceived value plays an important role in building brand loyalty.

Combined with the view that perceived value directly determines loyalty, many researchers put forward that consumers’ perceived value also indirectly affects the loyalty intensity of products or brands they are interested in through satisfaction. Specifically, Chiou (2004) and Lai et al. (2009) found that perceived value had a positive impact on overall satisfaction and loyalty intention, while overall satisfaction affected loyalty intention (Chiou, 2004; Lai et al., 2009). Similar findings have been reported in a variety of research settings, including online banking services (Yang and Peterson, 2004), hotels (Han et al., 2011), festivals (Yoon et al., 2010), restaurants (Tam, 2004), business-to-business services (Lam et al., 2004) and the cruise industry (Petrick, 2004b), as well as the wider service environment (Cronin et al., 2000). Therefore, perceived value not only has a direct impact on brand loyalty, but also can improve customer satisfaction, and then affect brand loyalty.

In addition, it has been proved that perceived value plays an intermediary role between perceived quality and brand loyalty. To support this relationship, Grewal and Krishnan (1998) accumulated insights based on their previous studies and other related studies reported in the literature (Parasuraman et al., 1985; Zeithaml, 1988; Dodds et al., 1991; Zeithaml et al., 1996; Grewal et al., 1998), developed a conceptual framework to clarify the general concept that quality of service enhances the perceived value and thereby loyalty. In addition, experimental studies show that the trade-off between perceived price and perceived quality leads to perceived value, and perceived value is the main factor determining purchase intention (Chang and Wildt, 1994). Some authors (Tam, 2004; Hollebeek and Brodie, 2009; Lai et al., 2009; Nam et al., 2011) provide strong evidence for the sequential chain of quality, value and loyalty, in addition to similar findings reported by Grewal et al. (1998).

The research of Jarvenpaa and Todd (1996) show that trust can offset the risk factors in the purchase environment, alleviate the uncertainty of transactions, thus reduce transaction costs and produce a cooperation-induced effect. The purpose of relationship marketing is to establish, maintain and strengthen customer relations. To ensure benefits, trust is very important in the mutual benefit relationship between buyers and suppliers. To improve trust, it is emphasized that the value awareness of suppliers must be improved. There is a view that the higher consumers’ perception of brand value, the higher their trust in the brand. Sultan et al. (2021) point out that when customers perceive the value of the products or services they consume, even if the value perception is not very high, customers will still have a high degree of trust in them. Kim et al. (2012) showed that consumers’ perceived value of low-cost airlines will affect consumers’ trust. Park and Jeon (2013) divided the perceived value of social media into functional value, emotional value, monetary value, information value, and social value, and confirmed that perceived value had a deliberate impact on trust. Kim et al. (2014) took the customers of private brand (PB) goods in large supermarkets as the object and studied the relationship between their perceived value of goods, brand trust, and purchase intention. To sum up, we can find that perceived value is an important variable that has an impact on trust. Thus, hypothesis 2b is as follows:

**H2b:** Perceived value has a direct positive impact on brand trust.

#### Customer Satisfaction

Customer satisfaction is one of the important factors affecting brand loyalty. Although most early researchers considered satisfaction as a cognitive structure (Olson and Dover, 1979; Oliver, 1980), recent definitions of satisfaction (Halstead et al., 1994; Spreng et al., 1996; Oliver et al., 1997; Olsen, 2002) seem to form a consensus, that is, the concept of satisfaction is an emotional structure, which recognizes the emotional response to product acquisition and consumption (Giese and Cote, 2000; Russell-Bennett and Bove, 2001). From this point of view, one of the most widely used definitions of customer satisfaction indicates that satisfaction is the degree to which consumers think that owning or using services can arouse positive emotions (Rust and Oliver, 1994).

The standard approach to conceptualizing the satisfaction-loyalty relationship assumes that the increase in loyalty comes from a higher level of satisfaction (Butcher et al., 2001). This positive relationship is based on the idea that consumers form satisfactory judgments about the products or brands they consume, which in turn explains why consumers are loyal to brands (Fullerton, 2005). Based on this reasoning, empirical studies have produced evidence supporting the positive impact of customer satisfaction on attitudinal loyalty (Macintosh and Lockshin, 1997; Jones and Suh, 2000; Butcher et al., 2001; Bennett et al., 2005; Rauyruen and Miller, 2007; Russell-Bennett et al., 2007; Li and Petrick, 2008; Sun et al., 2008; Yuksel et al., 2010; Han et al., 2011), behavioral loyalty (Yoon et al., 2010; Nam et al., 2011), and compound loyalty (Shankar et al., 2003; Rauyruen and Miller, 2007; Kim, 2011). Therefore, the view that customer satisfaction leads to brand loyalty is generally accepted.

Customer satisfaction is a positive emotional state formed by customers’ evaluation of products and enterprises, and it is the first variable of trust. Customer satisfaction is the initial relationship between the brand and the customer, and trust is the stage after customer satisfaction (Dwyer and Oh, 1987). In other words, satisfaction with the results will give the customer a sense of “being treated fairly,” which will convince the customer that the company cares about them and build trust in them (Ganesan, 1994). Customer satisfaction has a positive impact on trust (Ganesan, 1994; Garbarino and Johnson, 1999). Thus, hypothesis 2c is as follows:

**H2c:** Customer satisfaction has a direct positive impact on brand trust.

### Brand Trust

Brand trust is another common antecedent of brand loyalty. According to Moorman et al. (1992), trust is “a willingness to rely on an exchange partner in whom one has confidence” (p. 315). Trust leads to brand loyalty and commitment because it creates highly valued exchange relationships (Morgan and Hunt, 1994).

Loyalty and commitment are therefore the continuation and maintenance of valuable and important relationships created by trust (Chaudhuri and Holbrook, 2001). Theoretical reasoning about the relationship between trust and loyalty has identified three ways in which trust enhances an individual’s commitment to relationships (Ganesan, 1994; Ganesan and Hess, 1997). First of all, trust reduces the perceived risk level related to the opportunistic behavior of partners. Secondly, trust increases the confidence of partners that short-term inequalities will be solved for a long time. Finally, trust reduces the transaction cost in the exchange relationship. Consistent with this view, many studies have provided empirical evidence to show the contribution of trust to brand loyalty (Garbarino and Johnson, 1999; Chaudhuri and Holbrook, 2001; Sirdeshmukh et al., 2002; Ball et al., 2004; Chiou, 2004; Aydin et al., 2005; Flavián et al., 2006; Matzler et al., 2008; Cheng et al., 2012). Therefore, brand trust is an important prerequisite for customer loyalty to the brand.

Another concept of the trust-loyalty relationship assumes that trust mediates the positive impact of customer satisfaction on loyalty. Ravald and Grönroos (1996) explain this relationship, arguing that when consumers are satisfied, they begin to feel safe with suppliers, which leads to increased trust in suppliers and supports and encourages customer loyalty. Therefore, a satisfactory experience strengthens consumers’ trust in the organization. A highly satisfying experience cannot only convince consumers that trust in the organization is good, but also enhance that trust (Singh and Sirdeshmukh, 2000). Increased trust, in turn, leads to a long-term commitment to a relationship (Morgan and Hunt, 1994; Doney and Cannon, 1997; Garbarino and Johnson, 1999) and thus affects consumer loyalty to the brand. A series of studies have provided strong support for this sequential relationship (Singh and Sirdeshmukh, 2000; Delgado-Ballester and Munuera-Alemán, 2001; Shankar et al., 2003; Flavián et al., 2006; Caceres and Paparoidamis, 2007; Matzler et al., 2008; Van Doorn et al., 2010). The results of these studies and the theoretical reasoning provided in the literature support the significant chain effects of customer satisfaction trust and loyalty.

### Behavior Intention

Behavior intention, as a factor to predict consumer behavior, is regarded as the core element of relationship marketing. In addition, action intention is the main variable in many research fields. According to different research fields, it can be applied in various forms. The so-called action intention refers to the personal will and belief expressed by specific future behaviors after consumers form an attitude toward a certain object. Mainly reflected in price sensitivity of products/services, repurchase intention, WOM, etc. (Lounsbury and Polik, 1992). Frazier et al. (1989) argue that behavior intention is based on the relationship between customers and service personnel. Behavior intention refers to the knowledge generated after enjoying services, which is a stage in the decision-making process of consumers. It can also be called the evaluation process of satisfaction or dissatisfaction after enjoying the service. Ajzen (1991) claims that behavior intention is an intermediate variable between personal attitude and behavior, a subjective possibility when trust and attitude are behaviorized, and also a subjective state of individuals.

WOM is an essential variable for future actions (Kim et al., 2009). Previous studies have shown that the interaction between customers who do not merely share promotional information is an essential factor in customer decision-making (Feick and Price, 1987). WOM regarding products releases information related to the consumer experience through various means, the key determinant of good communication (Nie et al., 2019). WOM communication is also crucial for enterprises because customers evaluate the products and others’ feelings before choosing them (Belanche et al., 2020). Consequently, customers’ WOM is usually more attractive than other forms of communication (Dessart et al., 2018). Some authors (Siqueira et al., 2019) identified the positive effect of customer experience on WOM behavior. Chattopadhyay and Laborie (2005) found that if customers were satisfied with the service experience, they recommended it to their friends and intended to experience it again. Recommendation or WOM communication has become one of the most effective marketing tools. Comments on social media can affect the financial performance of other brands (Pansari and Kumar, 2017).

Over the past decade, customer engagement has been a pioneering study of brand loyalty and customer purchases (Prentice and Loureiro, 2017). When a customer experiences a brand, a strong psychological connection is formed (Hapsari et al., 2016), leading to customers’ repeated purchases or use of products of the brand. Customer engagement may establish a long-term relationship with the brand (Vivek et al., 2012). Previous studies have shown that the consumption experiences of customers have a significant impact on future repurchase behavior. Gounaris et al. (2007) highlighted that reuse intention is the core concept for maintaining the continuous relationship between brands and customers. In terms of reuse intention, the expected benefits come from experience mainly. Schivinski and Dabrowski (2014) also showed that customers’ communication experience on-brand social media pages impacted brand purchase intention significantly. Experience has a positive effect on customer attitude, affecting customer satisfaction. Experienced customers tend to have better satisfaction and a more positive attitude than inexperienced customers (Cheng et al., 2012). Previous studies have confirmed the influence of experience on the relationship between customer satisfaction and customer reuse intention (Khalifa and Liu, 2007).

Zhou et al. (2009) in the study of online purchasing behavior show that the service quality of websites has a stronger impact on trust and satisfaction than design quality. Trust has an impact on repurchase intention. Chu et al. (2012) think that for retail customers, the exploratory research on service quality, satisfaction, trust, and store loyalty shows that service quality has a positive impact on customer satisfaction and trust, and customer satisfaction and trust have an impact on customer loyalty. Chen and Chen (2010) showed that service quality had a positive impact on trust, and trust has an intermediary effect on repurchase intention. Chou et al. (2011) proved that service quality had a positive impact on customer satisfaction and trust, and customer satisfaction and trust had a positive impact on the repurchase. The research on the golf driving range also shows that trust has a positive impact on repurchase intention (Yu et al., 2005). So many empirical studies show that trust affects repurchase intention. Based on the above research on brand trust and behavior intention, this study predicts the following:

**H3a:** Brand trust has a direct positive impact on WOM intention.
**H3b:** Brand trust has a direct positive impact on reuse intention.

### The Mediating Effect of Customer Brand Trust

Trust, a key concept in long-term brand relationships (Morgan and Hunt, 1994; Garbarino and Johnson, 1999), has been retained in this research. In their three-dimensional conceptualization of brand trust, Gurviez and Korchia (2003) consider that credibility, integrity, and benevolence relate to the paradigm of exchange. Credibility refers to an assessment of the partner’s ability to meet the terms of the exchange, the expected performance, leading to functional expectations being achieved and needs satisfied. Integrity refers to the assignment of fair incentives to the partner relating to the fulfillment of its promises in terms of trade. These two facets are therefore mostly cognitive. Finally, benevolence focuses on sustainability and therefore the prospect of a less uncertain future, taking the consumer’s interests into account and leading to conditions for a fair exchange. To summarize, this conceptualization of trust emphasizes not just its main characteristic of evaluation but also its sustainability over time.

Brand trust is defined as “the willingness of the average consumer to rely on the ability of the brand to perform its stated function” (Chaudhuri and Holbrook, 2001, p. 82). The importance of trust has already been illustrated in sustainable relationships between the seller and buyer (Sahin et al., 2011). It is the trust that makes customers intimate with a company (Morgan and Hunt, 1994). Trust is created when a company promises to provide quality products to consumers and successfully meets the promise (Ahmed et al., 2011).

Scholars have demonstrated that trust is a key determinant of behavioral intentions. Consumers who trust a brand are more likely to maintain their reuse intention and trust the brand’s word of mouth. Many scholars have also reviewed the link between brand trust and behavioral intention (Aydin and Özer, 2005; Dehdashti et al., 2012), and they revealed that the most important antecedent of behavior intention was trust.

**FIGURE 1:**
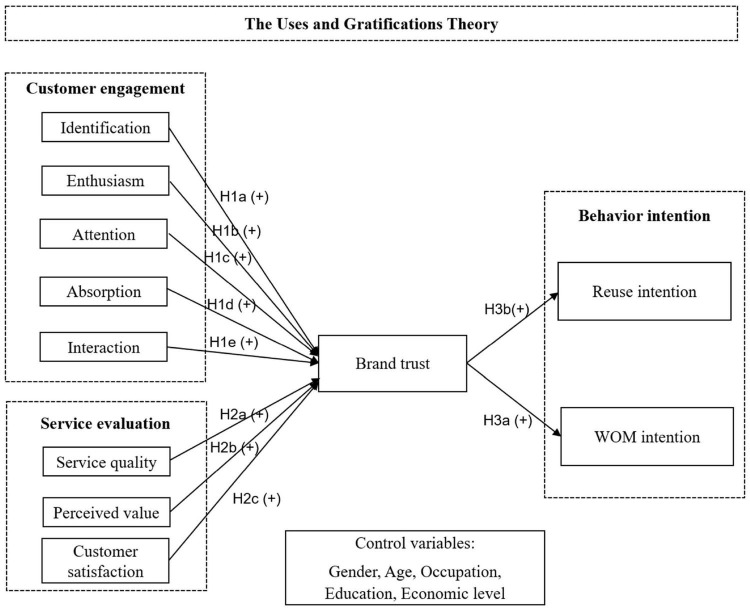
Conceptual research model.

This study primarily focuses on the major determinants of behavioral intention. Figure 1 illustrates the research framework for this study, showing the independent variables customer engagement and service evaluation, brand trust as a mediator variable, and behavior intention as the dependent variable. Hence, this study proposes the following hypothesis:

H4a: Brand trust plays a mediating role in the influence of customer engagement on behavior intention.
H4b: Brand trust plays a mediating role in the influence of service evaluation on behavior intention.

### Control Variable

This study introduced demographic characteristics as the primary control variables to further improve the model construction and the scale’s external validity. For example, the attention differences between men and women when choosing economy hotels and that young people are more likely to have access to choose different hotels than other groups. Customers with different occupations and economic levels have different evaluations of the hotel, which affects consumers’ WOM intention and reuse intention. The higher the level of education, the stronger the dependence on the used economy hotel brand. Thus, gender, age, occupation, education, economic level in demographic characteristics were selected as control variables in this study.

## Materials and Methods

### Theoretical Model

This study examines the impact of customer engagement and service evaluation on customers’ behavior intention. It tests whether the components of customer engagement, identification, enthusiasm, attention, absorption, and interaction positively impact trust and whether the components of service evaluation, service quality, perceived value, and customer satisfaction positively impact trust. Moreover, it examines whether trust positively influenced customers’ WOM intention and reuse intention. Figure 1 presents the conceptual research model.

### Instrument

Based on a large amount of domestic and international literature, the measurement of variables was based on the research results of domestic and international scholars. This study adopted some procedures to minimize the deviation of standard methods. First, the wording of items and questions should avoid ambiguity, be concise and straightforward, and ensure no unfamiliar terms and complex grammar. Second, the physical distance between the same construct measures is considered, not adjacent to the exact construction items.

The questionnaire was based on the Likert 7-point scale, with each measure ranging from “completely disagree” to “completely agree” on a scale of 1–7. To ensure content validity, the items used to measure the constructs were adapted from extensive literature and modified to fit the study context. Measurement items for customer engagement were adapted from So et al. (2013), with 15 question items; measurement items for service quality were adapted from Oh and Kim (2017), with 6 items; measurement items for perceived value were adapted from Yang and Peterson (2004), with 6 items; measurement items for customer satisfaction were adapted from Kim et al. (2018), with 4 items; measurement items for trust were adapted from Chaudhuri and Holbrook (2001), with 3 items; measurement items for WOM intention were adapted from Seo et al. (2020), with 3 items. Reuse intention was measured using four items adapted from Park and Park (2017).

### Data Collection

Before the official questionnaire was distributed, a small-scale pretest was conducted, and according to the results of the pretest, certain statements in the questionnaire were adjusted appropriately to form the official questionnaire.

The formal questionnaire was distributed from January to February 2021. The research data were collected by random sampling through online and offline surveys. Part of the data was collected directly near the economy hotel, and customers coming out of the hotel were randomly approached. Another part of the data was conducted through online crowdsourcing platform of China, functioning similarly to Amazon’s Mechanical Turk. Each participant’s Internet protocol address and demographic information were tracked and examined to ensure they submitted only one response. Questionnaires were administered to 470 customers who had used economy hotels, and finally, 437 questionnaires were used by setting the corresponding questionnaire validity as the censoring criteria, except for 33 questionnaires that were not suitable for this study.

Table 1 summarizes the demographic characteristics of the participants. Among the valid data collected, 44.9% were male and 55.1% were female, and the majority (63.84%) of the participants were between 20 and 39 years of age. Most of the participants were employed (59.2%). Furthermore, 99.1% of the participants were generally educated to college level or above. And the majority (61.1%) of the participants were of medium economic level.

**TABLE 1:** Demographics of the survey respondents (*N* = 437).

Item	Characteristic	Number of samples	Percentage
Gender	Male Female	196 241	44.9 55.1
Age	20–39 40–59 60 or older	279 157 1	63.8 36.0 0.2
Occupation	Institution and civil servant Enterprise staff Individual management Professional Staff Teacher Student Others	43 75 15 11 115 130 48	9.8 17.2 3.4 2.5 26.3 29.7 11.1
Education	High school and below College degree or above	4 433	0.9 99.1
Economic level	Below average Middle level Above average	67 267 103	15.3 61.1 23.6

## Data Analysis and Results

### Reliability and Validity

It is necessary to test the measurement model and evaluate the structural model to verify the tool’s reliability and validity. Confirmatory factor analysis and reliability tests were applied. The test followed a two-step method recommended by Anderson and Gerbing (1988).

The data were processed and analyzed using the software SPSS 22.0 and AMOS 24.0, and the structural equation modeling two-step method was used to test the model and hypotheses. First, the Harman one-way test was used to test for homoscedasticity. After unrotated factor analysis of the 40 items of the questionnaire, 11 factors with a characteristic root greater than 1 were obtained, and the first factor loading only accounted for 30.127% of the total loading, which was below the critical value level, indicating that this study was influenced by homoscedasticity within an acceptable range. The results of the reliability test showed that the overall Cronbach’s alpha value was 0.924, the Cronbach’s alpha value for each variable was higher than 0.920 (0.7), and the degree of the combination was greater than 0.860, which was higher than the minimum critical level of 0.6, so the scale had good reliability.

Factor analysis was used to test the convergent validity, and the overall sample had a KMO test value of 0.921 and a spherical Bartlett’s test chi-square value of 244459.242 (*p* < 0.001), which is suitable for factor analysis. Principal component analysis was used to test each construct, the maximum variance method was chosen for factor rotation, and the factor extraction method was eigenvalues greater than 1. The results showed that the factor loadings of the question items in the same construct were greater than 0.5, indicating that the convergent validity of the scale was good. Convergent validity was discriminated by the CR value of composite reliability and the average variance extracted (AVE), and the results showed that the criteria of composite reliability greater than 0.7 and AVE value greater than 0.5 were passed; Discriminant validity is to compare the individual mean-variance extracted values of the two constructs with the correlation coefficient between the two constructs, if the mean-variance extracted values of both constructs are greater than the squared correlation coefficients of the two construct variables, it means that there is good discriminant validity between the constructs, and the results show that the discriminant validity meets the requirements, i.e., the correlation coefficient matrix values are less than the diagonal AVE values. The correlation analysis matrices between the dimensions are shown in Tables 2, 3.

**TABLE 2:** Results of confirmatory factor analysis.

Construct	Measurement item	Standard loading	AVE	CR	Cronbach’s α
Identification	IDE1	0.959	0.842	0.941	0.972
	IDE2	0.961			
	IDE3	0.962			
Enthusiasm	ENT1	0.958	0.831	0.937	0.969
	ENT2	0.957			
	ENT3	0.954			
Attention	ATT1	0.953	0.821	0.932	0.966
	ATT2	0.953			
	ATT3	0.950			
Absorption	ABS1	0.963	0.845	0.942	0.974
	ABS2	0.963			
	ABS3	0.965			
Interaction	INT1	0.961	0.821	0.932	0.971
	INT2	0.950			
	INT3	0.967			
Service quality	QUA1	0.956	0.826	0.966	0.985
	QUA2	0.956			
	QUA3	0.962			
	QUA4	0.956			
	QUA5	0.959			
	QUA6	0.958			
Perceived value	VAL1	0.961	0.831	0.967	0.985
	VAL2	0.961			
	VAL3	0.957			
	VAL4	0.959			
	VAL5	0.958			
	VAL6	0.957			
Customer satisfaction	SAT1	0.965	0.848	0.957	0.982
	SAT2	0.968			
	SAT3	0.965			
	SAT4	0.965			
Brand trust	TRU1	0.790	0.697	0.873	0.871
	TRU2	0.806			
	TRU3	0.793			
Reuse intention	REU1	0.814	0.745	0.898	0.860
	REU2	0.832			
	REU3	0.816			
WOM intention	WOM1	0.825	0.743	0.897	0.860
	WOM2	0.821			
	WOM3	0.813			

*IDE, Identification; ENT, Enthusiasm; ATT, Attention; ABS, Absorption; INT, Interaction; QUA, Service Quality; VAL, Perceived Value; SAT, Customer Satisfaction; TRU, Brand Trust; REU, Reuse Intention; WOM, Word-of-Mouth intention.*

**TABLE 3:** Correlations matrix.

Variable	Mean	Variance	Correlation matrix										
			1	2	3	4	5	6	7	8	9	10	11
IDE	5.190	2.12	***1***										
ENT	5.262	2.05	0.166	***1***									
ATT	5.081	1.99	0.197	0.012	***1***								
ABS	5.160	2.27	0.105	0.019	0.178	***1***							
INT	4.993	2.39	0.231	0.083	0.282	0.230	***1***						
QUA	5.050	2.19	0.134	0.022	0.258	0.032	0.108	***1***					
VAL	5.128	2.15	0.154	0.110	0.217	0.118	0.073	0.181	***1***				
SAT	5.331	2.38	0.126	0.016	0.096	0.195	0.194	0.144	0.172	***1***			
TRU	5.367	0.60	0.373	0.113	0.508	0.238	0.375	0.445	0.416	0.386	***1***		
WOM	5.362	0.56	0.357	0.213	0.469	0.374	0.352	0.427	0.412	0.459	0.810	***1***	
REU	5.376	0.55	0.277	0.182	0.436	0.368	0.376	0.385	0.380	0.422	0.850	0.919	***1***

*IDE, Identification; ENT, Enthusiasm; ATT, Attention; ABS, Absorption; INT, Interaction; QUA, Service Quality; VAL, Perceived Value; SAT, Customer Satisfaction; TRU, Brand Trust; WOM, Word-of-Mouth intention; REU, Reuse Intention. Bold values are self-correlated, with a correlation coefficient of 1.*

After testing the validity and reliability of the measurement, this study tests the hypothesis proposed by AMOS 24.0. Table 4 shows the model fitting index’s actual and recommended values obtained after the original model is modified. These data prove that the model’s appropriate index is better than the recommended threshold, showing that the model fits the data well.

**TABLE 4:** Measures of the model fit.

Fit index	X^2^/df	RMSEA	SRMR	GFI	CFI	IFI	TLI
Recommended range	<3^a^	<0.05^b^	0.05	>0.90^a^	>0.90^a^	>0.90^a^	>0.90^a^
Model value	1.304	0.026	0.043	0.909	0.992	0.992	0.990

*RMSEA, root mean square error of approximation; GFI, the goodness of fit index; CFI, comparative fit index; TLI, non-normed fit index.*

*^a^According to Bentler and Bonett (1980).*

*^b^According to Browne and Cudeck (1989).*

The validation factor analysis of the model using AMOS 24.0 yielded the fit indices: χ^2^/df = 1.304 < 3, GFI = 0.909, RMSEA = 0.026 < 0.05, SRMR = 0.043 < 0.05, TLI = 0.990, CFI = 0.992, IFI = 0.992, all of which were greater than 0.9, and the absolute and relative fit indices are within the acceptable range. Therefore, the collected sample data can be analyzed by structural equation modeling.

### Hypotheses Testing

**FIGURE 2:**
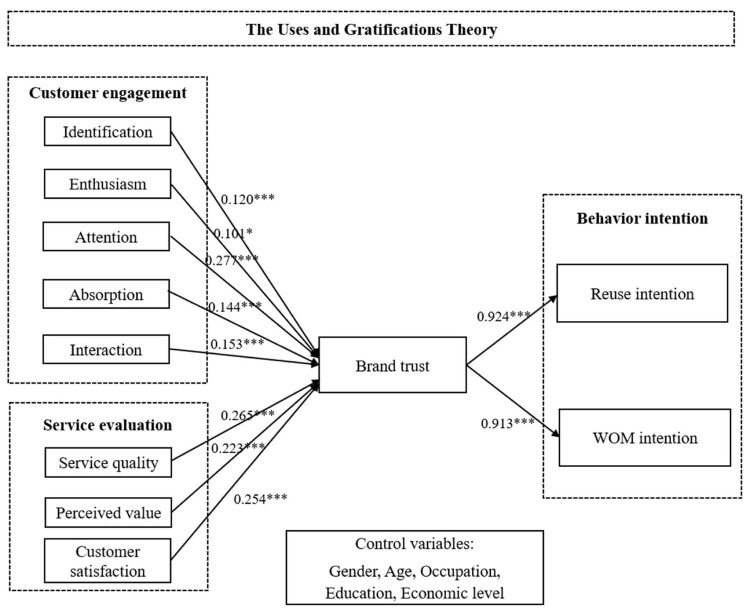
Path analysis results. Note. *p < 0.05; ***p < 0.001.

Path modeling was performed to test H1a to H3b. The results show that customer engagement (β = 0.130, *p* < 0.001), and service evaluation (β = 0.131, *p* < 0.001) positively affected trust. Therefore, H1 and H2 are supported. This study believes that these findings can explain how customer engagement and service evaluation affect customers’ behavior intention. Trust positively affect WOM intention (β = 0.451, *p* < 0.001) and reuse intention (β = 0.425, *p* < 0.001). Thus, H3 is supported. Therefore, our conceptual model provides a reasonable explanation for the different customer behavior in intentions. The results are presented in Table 5 and Figure 2.

**TABLE 5:** Structural model results.

Hypotheses	Structural path	S.E.	*T*-value	Results
H1a	Identification →dBrand trust	0.120***	3.349	Supported
H1b	Enthusiasm→aBrand trust	0.101*	2.960	Supported
H1c	Attention →Brand trust	0.277***	7.229	Supported
H1d	Absorption→Brand Trust	0.144***	4.090	Supported
H1e	Interaction→Brand trust	0.153***	4.174	Supported
H2a	Service quality→Brand trust	0.265***	7.344	Supported
H2b	Perceived value →Brand trust	0.223***	6.244	Supported
H2c	Customer satisfaction→Brand trust	0.254***	7.002	Supported
H3a	Brand trust→WOM intention	0.913***	17.125	Supported
H3b	Brand trust →rReuse intention	0.924***	17.084	Supported

*p < 0.05; ***p < 0.001.

This study used a bootstrapping method to test the mediating effect and found that brand trust plays a mediating role in customer engagement on behavior intention. Table 6 shows that the indirect effect of brand trust on the relationship between customer engagement and behavior intention is significant with a 95% bootstrap confidence interval, excluding 0. This finding shows that brand trust mediates the influence of customer engagement on behavior intention.

**TABLE 6:** Analysis of mediating effect.

Independent variable	Mediator variable	Dependent variable	Effect	S.E.	Confidence interval	*p*-value
Identification	Brand trust	WOM intention	0.110	0.037	[0.021, 0.098]	0.003**
Enthusiasm			0.092	0.034	[0.017, 0.085]	0.012*
Attention			0.253	0.040	[0.091, 0.171]	0.005**
Absorption			0.131	0.037	[0.025, 0.098]	0.006**
Interaction			0.140	0.038	[0.029, 0.097]	0.004**
Identification		Reuse intention	0.111	0.037	[0.020, 0.090]	0.004**
Enthusiasm			0.093	0.034	[0.015, 0.083]	0.011*
Attention			0.256	0.038	[0.090, 0.162]	0.004**
Absorption			0.133	0.036	[0.026, 0.096]	0.004**
Interaction			0.141	0.038	[0.028, 0.088]	0.004**
Service quality		WOM intention	0.242	0.035	[0.083, 0.147]	0.003**
Perceived value			0.204	0.034	[0.062, 0.132]	0.002**
Customer satisfaction			0.232	0.042	[0.063, 0.140]	0.005**
Service quality		Reuse intention	0.245	0.034	[0.076, 0.139]	0.004**
Perceived value			0.206	0.034	[0.059, 0.121]	0.003**
Customer satisfaction			0.235	0.041	[0.063, 0.131]	0.004**
95% Bootstrap confidence intervals for the indirect effect						

*p < 0.05; **p <0.01.

### Control Variable Results

To improve the scale’s external validity and ensure the scientificity of the verification results, gender, age, occupation, education, economic level in demographic characteristics were selected as control variables in this study. According to the study results, age, occupation and economic level have no significant impact on latent variables because consumers of different ages and occupations and economic levels may have little difference in the choice of economy hotel brands. Gender has a considerable effect on reuse intention (β = 0.096, *p* < 0.05), but has no significant effect on other latent variables. Generally speaking, men are more rational than women, so when choosing economy hotels, they tend to choose hotels that have been used and satisfied before. Women may be more emotional and like to try different hotels. Therefore, gender significantly impacts reuse intention. Finally, the education had a significant impact on WOM intention (β = 0.136, *p* < 0.01) and reuse intention (β = 0.088, *p* < 0.05) and had no significant impact on other latent variable. The education can explain consumers’ dependence on the brand of economy hotel and has different effects on WOM intention and reuse intention. In the future, demographic characteristics should be considered as moderating variables to modify and improve this study’s conclusions further.

## Discussion and Conclusion

### General Discussion

The hotel industry has been greatly affected by the New Crown epidemic. In this study, 437 hotel customers were selected to study the effects of customer engagement, service evaluation on brand trust and consumer behavioral intention, taking Chinese economy hotel companies as an example.

First, the findings show that customer engagement has a direct positive effect on brand trust, which is consistent with past literature (e.g., Hollebeek, 2011; Sashi, 2012; Vivek et al., 2012). Unlike previous studies, in this study, customer engagement was subdivided into identification, enthusiasm, attention, absorption and interaction, and the effects of these five dimensions on brand trust were investigated separately. The results show that identification, enthusiasm, attention, absorption, and interaction all have a direct positive effect on brand trust. Among them, the factor loadings of attention and interaction are higher, indicating that hotel companies are more able to gain consumers’ trust in the brand by paying attention to them, and hotel companies are more able to gain consumers’ trust in the brand when they have a certain level of interaction with them.

Secondly, the findings show that service quality, perceived value and customer satisfaction, all components of service evaluation, also have a direct and positive impact on brand trust. These results are consistent with existing studies in the literature, such as Liu (2020) who verified that dealer service quality has a positive effect on product brand trust. Zhong (2020) who verified that customer perceived value of AI products has a positive effect on product brand trust, and Li (2019) who verified that customer satisfaction has a positive effect on brand trust in luxury parent brands impact.

Thirdly, the results show that brand trust has a positive effect on consumer behavioral intention (reuse intention and WOM intention). This is consistent with previous studies (Yasin et al., 2020; Zhang et al., 2021).

Finally, unlike previous studies, this study set the mediating variable brand trust and explored the mediating effect of brand trust on the relationship between the five dimensions of customer engagement (identification, enthusiasm, attention, absorption, and interaction) and customer behavioral intentions (i.e., reuse and WOM). The results show that brand trust mediates the relationship between customer engagement (identification, enthusiasm, attention, absorption, and interaction) and customer behavioral intention. Moreover, this study also explored the mediating effect of brand trust on the relationship between three dimensions of service evaluation (service quality, perceived value, and customer satisfaction) and customer behavioral intention (reuse and WOM).

Therefore, economy hotels can enhance customers’ trust in the hotel by improving the service quality of the hotel, the perceived value of the hotel and customer satisfaction, which in turn increases customers’ WOM intentions and reuse intentions. Economy hotels should take active measures, such as actively participating in public welfare and environmental protection activities, to enhance customers’ perceptions of the company and increase consumers’ interest in the company. In addition, they can also encourage customers to contact the company by initiating some discussions on the company’s homepage and organizing offline parties among members to enhance consumers’ participation in company interaction online and offline.

### Theoretical Implications

With the rapid development of platform economy and social media, the interaction and cooperation between customers and enterprises are in full swing, customers become one of the main bodies of value co-creation (So et al., 2013), and highly compatible customers become the biggest hidden assets of enterprises. This idea urges service brands to adopt a customer engagement strategy to manage customer relationships more and more, which makes the concept of customer engagement become an important field of academic and practical circles in recent years. Despite this concern and the increasing assumption that it is closely related to the contact between potential and existing customers, the study of customer engagement from a psychological perspective is still in the initial stage of development. Although some researchers have emphasized the potential value of customer engagement (Patterson et al., 2006; Hollebeek and Brodie, 2009; Van Doorn et al., 2010; Brodie et al., 2011), empirical research on the components of customer engagement and how to measure this concept is very limited.

The marketing literature argues that customer engagement is a strategic sine qua non for establishing, maintaining, and strengthening positive long-term customer-brand relationships (Xie and Peng, 2010). However, up to now, there is no meaningful measurement mechanism that can be used to test this assertion empirically. This study provides a theoretically reasonable scale for marketing, which can be used by scholars to further expand their understanding of customer engagement. From a theoretical point of view, the Customer Participation Scale empirically studies the potentially related factors of customer participation, which provides a basis for building future knowledge of customer participation and expanding theoretical understanding of the concept of customer participation. For example, the most important factors affecting customer participation include attitude antecedents, such as brand attachment, brand commitment, and brand performance perception (Van Doorn et al., 2010). In addition, a conceptual model shows participation and interaction as antecedents of customer participation (Hollebeek and Brodie, 2009). Using the customer engagement scale proposed in this study, future studies can now empirically test these potential connections.

Moreover, the basic contribution of this study is to provide a theoretical basis and empirical evidence to support the relationship between the emerging concept of customer engagement and the key elements in the development of customer behavior intention. Although previous studies have supported the contribution of purchase-related factors, such as service quality and customer satisfaction, in building a strong service brand (Clemes et al., 2010), the findings of this study prove empirically that customer engagement exceeds purchase on WOM intention and repurchase also have a strong influence, making incremental contributions to existing knowledge.

### Management Implications

In addition to the theoretical contribution, this study also provides some practical enlightenment for the practice of brand management. The development and verification of customer engagement scale provide a valuable tool for the operation of economy hotels during the epidemic period, which can effectively measure the contact with customer groups and develop marketing strategies reasonably. Managers can use this scale to collect insightful information. For example, they can evaluate their brand’s performance in competition by comparing their customers to the customers of competing hotel brands. Moreover, because the scale developed in this study is a result measure, economy hotel managers can use it to verify various relationship marketing initiatives. Such insight will help managers determine whether they need to modify or change their marketing plan to achieve the desired goals.

This study shows that these five dimensions of customer engagement are very important in representing customer engagement. This result shows that when trying to develop customer engagement, economy hotel managers can focus on improving each of the five dimensions of customer engagement, with special emphasis on attention and interaction, because of their high factor load. For example, to increase attention, managers need to provide information that their customer base finds relevant and interesting (Celsi and Olson, 1988). Interaction is also an important dimension of customer engagement. To increase customer interaction, hotels need to provide customer interaction opportunities and incentives to encourage customer participation, such as identification and reward programs (Sawhney et al., 2005). Generally speaking, these actions help customers immerse themselves in the interactive experience with the brand, thus promoting their interaction with the brand. Although customer engagement is manifested beyond service transactions, excellent service, customer exciting functions, and good brand image may enhance customer enthusiasm for the brand (Bhote, 1995). In establishing a strong customer brand identification, brand managers must create unique and clear logos expected by target customer groups, because logos allow sustainable product differentiation and help enhance customer brand identification (Baumgarth and Schmidt, 2010).

Although managers can disseminate hotel-related information through many channels (such as Ctrip and Public Comment), this study shows that high-participation customers of hotels tend to participate in activities on hotel official websites, but are less likely to participate in activities on third-party websites. Given their ability to provide objective and enlightening product information, these third-party sites are often considered the first point of access to product information when customers need to make immediate purchase decisions. However, the information needs of active customers are based on their close connection with the brand, so they seek hotel information to satisfy their interest in the hotel. Therefore, the official website of the hotel provides participating customers with a more direct way to obtain information.

### Limitations and Future Research

This study explores the effects of customer engagement and service evaluation on consumer trust and behavior intention.

The results of the study have some practical reference value for airlines to enhance the trust of enterprises and promote customers’ behavior intention through customer engagement and service evaluation and have certain guiding significance for the marketing management of economy hotels. However, due to the limitation of time, money, and energy, this study inevitably has some limitations.

Firstly, using the survey as a data collection method may introduce measurement errors into research data. This measurement error may not only come from the scale used to measure the structure (Bennett et al., 2005), may also come from respondents’ inability to accurately report their past experience with economy hotel brands. However, by following a systematic and rigorous scale development process to verify customer engagement metrics, and by carefully considering the choice of reliable metrics of other structures tested in other empirical studies, the measurement errors of the scale are minimized. In addition, a thorough examination of the reliability and validity of the measurement structure produces strong evidence that the measurement scale has good psychological measurement characteristics, so it shows that the measurement error is not the main problem of this study.

Secondly, in the whole study sample, comparing the demographic characteristics of the sample with those of the general population shows that the respondents are different in several demographic variables (such as age and occupation). Therefore, the sample may not fully represent the general population.

Thirdly, this study did not distinguish the role of research variables before, during and after COVID-19. The different period may have an impact on the study.

Several possible areas for future research on this research are put forward. First of all, because this study only carried out a sample survey of hotel customers, it is necessary to further test the scale and proposed model of customer engagement in other service environments such as health care, aviation, and banking in future research. This test will make the research results more universal in other service environments, and further, understand the degree to which the model explains the formation of customer behavior intention of service brands in different service environments.

Another possible area of future research involves negative customer participation. Consistent with the discussion of customer engagement in the literature, this study investigates customer engagement from a positive perspective. However, some literature shows that customer engagement can also be expressed as negative prices, such as anti-brand activities (Van Doorn et al., 2010). Therefore, future research should explore various forms of negative customer engagement behaviors or expressions and how they affect the results of customer engagement.

Future research can also expand and test the proposed research model, including other factors that may represent the antecedents and outcomes of customer engagement. For example, in the qualitative stage of this study, five main factors affecting customer engagement activities are identified. These factors can be incorporated into the research model and tested in subsequent quantitative studies to determine their relationship with customer engagement. Similarly, the literature on customer engagement shows that customer engagement may influence aspects such as brand awareness, customer loyalty, customer assets, etc. (Van Doorn et al., 2010). To further improve consumers’ WOM and repurchase behavior intention, future research can investigate the influence of customer engagement on these factors.

## Data Availability Statement

The raw data supporting the conclusions of this article will be made available by the authors, without undue reservation.

## Ethics Statement

Ethical review and approval was not required for the study on human participants in accordance with the local legislation and institutional requirements. Written informed consent from the patients/participants was not required to participate in this study in accordance with the national legislation and the institutional requirements.

## Author Contributions

XC was responsible for writing the manuscript. XL was responsible for data collection. YW was responsible for proofreading English. JZ was responsible for literature collation. All authors contributed to the article and approved the submitted version.

## Conflict of Interest

The authors declare that the research was conducted in the absence of any commercial or financial relationships that could be construed as a potential conflict of interest.

## Publisher’s Note

All claims expressed in this article are solely those of the authors and do not necessarily represent those of their affiliated organizations, or those of the publisher, the editors and the reviewers. Any product that may be evaluated in this article, or claim that may be made by its manufacturer, is not guaranteed or endorsed by the publisher.
